# Two dimensional inorganic electride-promoted electron transfer efficiency in transfer hydrogenation of alkynes and alkenes†
†Electronic supplementary information (ESI) available: Experimental details and characterization. See DOI: 10.1039/c5sc00933b


**DOI:** 10.1039/c5sc00933b

**Published:** 2015-04-13

**Authors:** Ye Ji Kim, Sun Min Kim, Eun Jin Cho, Hideo Hosono, Jung Woon Yang, Sung Wng Kim

**Affiliations:** a Department of Energy Science , Sungkyunkwan University , Suwon 440-746 , Korea . Email: kimsungwng@skku.edu ; Email: jwyang@skku.edu; b Center for Integrated Nanostructure Physics , Institute for Basic Science , Sungkyunkwan University , Suwon 440-746 , Korea; c Department of Chemistry , Chung-Ang University , Seoul 156-756 , Korea; d Materials and Structure Laboratory , Tokyo Institute of Technology , Yokohama 226-8503 , Japan

## Abstract

A simple and highly efficient transfer hydrogenation of alkynes and alkenes by using a two-dimensional electride, dicalcium nitride ([Ca_2_N]^+^·e^–^), as an electron transfer agent is disclosed. Excellent yields in the transformation are attributed to the remarkable electron transfer efficiency in the electride-mediated reactions. It is clarified that an effective discharge of electrons from the [Ca_2_N]^+^·e^–^ electride in alcoholic solvents is achieved by the decomposition of the electride *via* alcoholysis and the generation of ammonia and Ca(O^i^Pr)_2_. We found that the choice of solvent was crucial for enhancing the electron transfer efficiency, and a maximum efficiency of 80% was achieved by using a DMF mixed isopropanol co-solvent system. This is the highest value reported to date among single electron transfer agents in the reduction of C–C multiple bonds. The observed reactivity and efficiency establish that electrides with a high density of anionic electrons can readily participate in the reduction of organic functional groups.

## Abstract 2

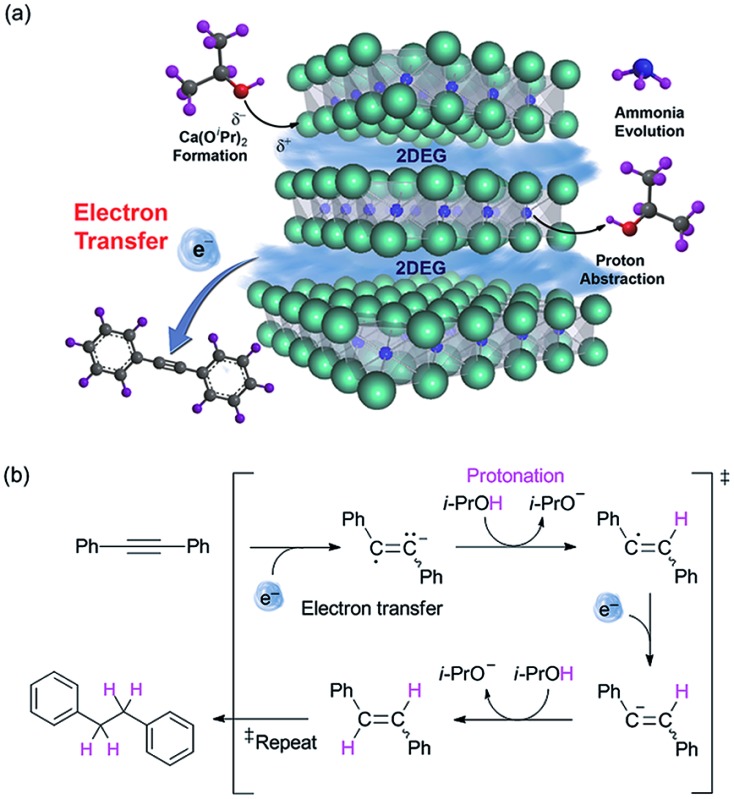
A simple and highly efficient transfer hydrogenation of alkynes and alkenes by using a two-dimensional electride, dicalcium nitride ([Ca_2_N]^+^·e^–^), as an electron transfer agent is disclosed.

## Introduction

The development of simple and efficient chemical transformation routes with maximal yields has been a continuously pursued challenge in synthetic chemistry. Such protocols can provide important benefits in the field of organic synthesis such as saving starting materials, reagents, and energy, thereby lowering production costs and environmental impacts.^1^ Among fundamental reactions in synthetic organic chemistry, the reduction of organic functional groups with carbon–carbon (C–C) multiple bonds is one of the most universally applied and crucial synthetic processes in academic and industrial circles.^2^ The reduction of C–C multiple bonds has been widely used in the synthesis of natural products and pharmaceutical compounds as well as in petroleum chemistry.^3^ The established methods for this are (1) hydrogenation, where hydrogen gases are activated on transition metal catalysts such as Pt, Ru, Pd, Rh or Ni; and (2) transfer hydrogenation, where hydrogen from source materials is added to a substrate.^4^,^5^ Transfer hydrogenation can be further classified into three kinds of methods based on the initiation mode: (1) electron transfer, (2) hydride transfer, and (3) hydrogen atom transfer.^6^ Among these reactions, the electron transfer method using an electron donating agent is currently the most classical and general reaction in synthetic chemistry.

For efficient electron transfer hydrogenation, it is essential to use an agent with a high reduction potential to facilitate the electron transfer. In this regard, many types of materials, such as simple metals (Mg and Yb), stabilized alkali metal systems (Na in silica-gel and in ammonia liquid), and lanthanide iodides (SmI_2_ and TmI_2_), have been employed in electron transfer hydrogenation.^7^ Despite their effectiveness in the reported transfer hydrogenations, there are several drawbacks in the methodology. Major disadvantages include the toxicity, the cost of agents and the rigorous reaction conditions. Furthermore, the separation of products from the resultants is laborious and inefficient, yielding pollutants. Of all these drawbacks, the low electron transfer efficiency of the reaction is the most critical issue to be addressed for efficient transfer hydrogenation. For example, simple metals show a limited efficiency of 30%. Although moderate electron transfer efficiency in SmI_2_-mediated reactions has been achieved, the use of toxic and rare lanthanide elements, and the requirement of additives such as amines, are drawbacks that need to be resolved.

In order to achieve simple and efficient electron transfer hydrogenation for C–C multiple bonds, it is necessary to use a strong electron transfer agent and to simplify the reaction process, satisfying the following characteristics. First, the material must have a sufficient reduction potential to effectively transfer the electrons to the C–C multiple bonds. Second, the material must consist of abundant elements to replace the high cost of lanthanides and circumvent the further purification process. Third, the reaction process must be simple to directly transfer the electrons from the source agents to the substrates. Fourth, the reaction must occur under very mild conditions without forming side products, which consume the electrons of the agents in the transferring process. In this regard, the use of electrides in alcoholic solvents meets the four requirements with respect to their potential for electron transfer based on low work functions and mild alcoholysis. Herein, we report a simple and highly efficient transfer hydrogenation of alkenes and alkynes by using an inorganic electride, [Ca_2_N]^+^·e^–^, as the efficient electron transfer agent in the alcoholic solvents. This protocol provided up to 80% electron transfer efficiency, which is the highest value among the electron transfer agents in the reductive reactions of C–C multiple bonds. From the reactivity control of the electride in alcoholic solvents, we demonstrated that the choice of solvent in terms of both acidity and polarity was crucial for maximizing the electron transfer efficiency. It is also suggested, from the working model based on the mechanistic studies through X-ray diffraction and ion chromatography, that the alcoholysis played a key role in transferring the anionic electrons of the electride.

Electrides are ionic crystals in which loosely trapped interstitial electrons in crystallographic empty space such as cavities, channels or interlayers behave as anions.^8^ The degree of interaction between trapped anionic electrons, *i.e.*, localization or delocalization, largely determines the chemical and physical properties of the system.^9^ The most characteristic feature of the electrides is their low work function based on the loosely trapped anionic electrons.^10^ The first crystalline electride, Cs^+^(18-crown-6)_2_·e^–^, was synthesized by using solvated electrons as precursors.8*c* The solvated electrons in an alkali metal–amine solution were successfully separated from the cations of alkali metals with the addition of crown ethers and occupied the structural cavities in the grown crystals of the electride. In synthetic chemistry, while solvated electrons as a potent reducing agent were applied to the classical Birch and Bouveault–Blanc reductions, organic electrides have not been used as functional materials due to their fatal drawback of thermal and chemical instability at room temperature.^11^

In contrast, the room temperature stable inorganic electride [Ca_24_Al_28_O_64_]^4+^·4e^–^ (C12A7:e^–^) mitigates the drawback of organic electrides, providing access to a new frontier in synthetic chemistry. As the inherent electrons in the electride can directly participate in chemical reactions, the use of an electride in synthetic chemical reactions would be energetically favourable compared to the electrochemical reactions, which use additional electrical energy with a low electron transfer rate, and indispensable noble metal electrodes and electrolytes. Moreover, as already demonstrated, this electride-mediated methodology is feasible for scalable reactions such as the pinacol coupling reaction using the [Ca_2_N]^+^·e^–^ electride.^12^

The C12A7:e^–^ electride was verified to be an effective electron donor in chemical reactions such as ammonia synthesis and decomposition, N_2_ dissociation, and CO_2_ splitting.^13^ Recently, a two-dimensional (2D) electride, [Ca_2_N]^+^·e^–^, which has anionic electron layers between the cationic framework layers ([Ca_2_N]^+^), was discovered.^14^ This 2D electride showed very promising characteristics as a strong electron donor for synthetic chemistry in terms of its small work function (2.6 eV) and high electron concentration (∼1.37 × 10^22^ cm^–3^), similar to those of typical alkali metals. As the open layer structured [Ca_2_N]^+^·e^–^ electride provides better accessibility to anionic electrons, when compared to the close cage structured C12A7:e^–^ electride, we conducted direct electron transfer from a C12A7:e^–^ electride to various organic substrates bearing C–C multiple bonds and demonstrated highly efficient electron transfer hydrogenation of alkynes and alkenes in alcoholic solvents.^12^,^15^

## Results and discussion

Initially, we examined the effects of the solvent as an electride activator and proton donor, and optimized the reaction conditions (Table 1). The use of a methanol solvent showed a low conversion of **1a** (Table 1, entry 1). The reaction rapidly proceeded with the sudden decomposition of the [Ca_2_N]^+^·e^–^ electride, forming a gel-type suspension and degrading the homogeneity of the reaction mixture. Since the methanol solvent reduces the reaction rate and the solubility of the starting materials, we applied a THF : MeOH co-solvent for a gentler reaction that can increase the solubility of the starting material and improve the homogeneity of the reaction mixture, leading to an increased conversion rate (Table 1, entry 2). Further optimization was investigated by increasing the equivalents of the electride (Table 1, entries 2–5). Whereas the yield of **1a′** increased as the equivalents of the electride increased, the electron transfer efficiency was moderate. We further postulated that both the acidity and the polarity of the solvent may influence the reactivity of the electride. Indeed, a significant enhancement in the yield of **1a′** was observed by switching the solvent from methanol to isopropanol (Table 1, entries 5–7). The electron transfer efficiency was improved with decreasing acidity of alcohols [p*K*_a_; methanol (15.1), ethanol (15.9), isopropanol (17.1)], although the reaction times were prolonged. This phenomenon is ascribed to the hydrogen evolution consuming electrons by reacting with the alcohols. A relatively acidic alcohol readily reacts with electrons to liberate hydrogen gas, consuming electrons and then reducing efficiency. Thus, employing less acidic alcohols such as isopropanol is essential to achieve the high yield of **1a′** due to the enhanced electron transfer efficiency.

**Table 1:** Optimization of transfer hydrogenation for alkyne utilizing [Ca_2_N]^+^·e^–^^*a*^

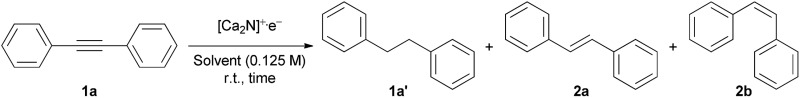						
Entry	[Ca_2_N]^+^·e^–^ (equiv.)	Time	Solvent	Conversion^*b*^	**1a′** : **2a** : **2b** ratio^*b*^	Efficiency^*e*^ (%)
1	2	6 h	MeOH	24%	70 : trace : 30	41
2	2	2 h	THF : MeOH (1 : 1)	40%	78 : 4 : 18	71
3	3	2 h	THF : MeOH (1 : 1)	54%	82 : 2 : 16	66
4	4	2 h	THF : MeOH (1 : 1)	70%	88 : trace : 11	66
5	5	2.5 h	THF : MeOH (1 : 1)	84%	97 : trace : 3	66
6	5	14 h	THF : EtOH (1 : 1)	93%	85 : trace : 14	69
7	5	24 h	THF : iPrOH (1 : 1)	>99%	79 : 9 : 12	72
8	5	15 h	Toluene : iPrOH (1 : 1)	85%	74 : trace : 26	59
9	5	15 h	DMF : iPrOH (1 : 1)	>99%(96%)^*c*^	100 : trace : trace	80
10^*d*^	5	15 h	DMF : iPrOH (1 : 1)	N.D.	N.D.	—

^*a*^Conditions: diphenylacetylene (0.5 mmol, 0.125 M), [Ca_2_N]^+^·e^–^ [1–2.5 mmol (2–5 equiv.)], r.t.

^*b*^Determined by gas chromatography.

^*c*^Isolated yield.

^*d*^[Ca_24_Al_28_O_64_]^4+^·4e^–^ electride was used instead of [Ca_2_N]^+^·e^–^.

^*e*^The electron transfer efficiency was calculated from the ratio of the participated electrons in the reactions to provide electrons from the electride.

Finally, we used a polar solvent as a co-solvent to maximize the yield of **1a′** and the electron transfer efficiency (Table 1, entry 9). The use of the polar solvent DMF with iPrOH led to significant enhancement in both the yield (>99%) and the electron transfer efficiency (∼80%). These results suggest that a more polar environment might enhance the solvation effect of anionic electrons from the electride in the co-solvent or the dissociation of ionic bonds between the cationic layers and the anionic electrons of the electride, leading to the high electron transfer efficiency. Indeed, a transient solvated electron in the water of the highest polar solvent is a well-known phenomenon in radiation chemistry, implying the possible existence of transient solvated electrons in the DMF–iPrOH solvent. In addition, no reaction occurred when the C12A7:e^–^ electride was employed under the optimized reaction conditions. This also supports the gentle alcoholysis of the layer structured [Ca_2_N]^+^·e^–^ electride in DMF–iPrOH solvent, which allows the efficient transfer of anionic electrons to substrates. However, we could not achieve a selective hydrogenation during the reaction due to the thermodynamic favour. The relative reactivity of aromatic substrates increases in the following order: *diphenylacetylene* ≪ *cis-stilbene* ≈ *trans-stilbene*, indicating the difficulty of selective hydrogenation for *cis*- and *trans*-stilbene from diphenylacetylene.^16^

We also examined the electron transfer hydrogenation of alkenes in this protocol using *trans*-stilbene **2a** as a model compound. Similar to the results of alkyne, the use of a co-solvent improved the conversion of **2a** (Table 2, entries 1–3). In addition, the relationship between the acidity of the solvents and the electron transfer efficiency was identical (Table 2, entries 3–5), strongly suggesting that the rate of the protonation step is an important parameter for enhancing the electron transfer efficiency. It is notable that the reaction with DMF : MeOH was completed in one hour, providing a quantitative yield of **1a′**.

**Table 2:** Optimization of transfer hydrogenation for an alkene utilizing [Ca_2_N]^+^·e^–^^*a*^

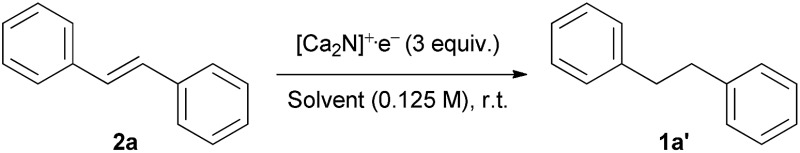			
Entry	Time	Solvent	Conversion^*b*^
1	1 h	MeOH	62%
2	1 h	Toluene : MeOH (1 : 1)	75%
3	1 h	DMF : MeOH (1 : 1)	>99%
4^*c*^	2 h	DMF : EtOH (1 : 1)	79%
5^*c*^	14 h	DMF : iPrOH (1 : 1)	35%

^*a*^Conditions: *trans*-stilbene (0.5 mmol, 0.125 M), [Ca_2_N]^+^·e^–^ (1.5 mmol, 3 equiv.), r.t. solvent.

^*b*^Determined by gas chromatography.

^*c*^[Ca_2_N]^+^·e^–^ remained after reaction.

With the optimized conditions in hand, the substrate scope of alkynes and alkenes was examined (Table 3). Both electron-rich and electron-deficient aromatic alkynes were well converted into the corresponding alkanes with excellent yields (Table 3, entries 1–8). Terminal alkyne **1i** was also a suitable substrate for electron transfer hydrogenation, showing an excellent yield (Table 3, entry 9). Reactions of stilbene derivatives also proceeded well, providing excellent yields of the corresponding alkanes, indicating the possibility for much broader applications of the present protocol (Table 3, entries 10–14).

**Table 3:** Scope of transfer hydrogenation for alkynes and alkenes utilizing [Ca_2_N]^+^·e^–^^*a*^

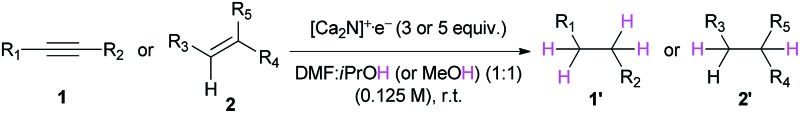				
Entry	Substrate	Product	Time	Yield^*b*^
1	R_1_ = R_2_ = C_6_H_5_ (**1a**)	**1a′**	15 h	96%
2	R_1_ = 2-Me-C_6_H_4_, R_2_ = C_6_H_5_ (**1b**)	**1b′**	21 h	89%
3	R_1_ = 3-OMe-C_6_H_4_, R_2_ = C_6_H_5_ (**1c**)	**1c′**	21 h	95%
4	R_1_ = 4-OMe-C_6_H_4_, R_2_ = C_6_H_5_ (**1d**)	**1d**′	10 h	98%
5^*c*^	R_1_ = R_2_ = 4-Me-C_6_H_4_ (**1e**)	**1e′**	24 h	97%
6	R_1_ = 4-F-C_6_H_4_, R_2_ = C_6_H_5_ (**1f**)	**1f′**	12 h	96%
7	R_1_ = 2,4-F-C_6_H_4_, R_2_ = C_6_H_5_ (**1g**)	**1g′**	14 h	93%
8	R_1_ = 4-F-C_6_H_4_, R_2_ = 4-F-C_6_H_4_ (**1h**)	**1h′**	24 h	94%
9	R_1_ = 4-Ph-C_6_H_4_, R_2_ = H (**1i**)	**1i′**	42 h	92%
10^*d*^	R_3_ = R_4_ = C_6_H_5_, R_5_ = H (**2a**)	**1a′**	1 h	99%
11^*d*^	R_3_ = C_6_H_5_, R_4_ = H, R_5_ = C_6_H_5_ (**2b**)	**1a′**	1 h	97%
12^*d*^	R_3_ = R_4_ = C_6_H_5_, R_5_ = C_6_H_5_ (**2c**)	**1c′**	1 h	92%
13^*d*^	R_3_ = 3,5-OMe-C_6_H_3_, R_4_ = C_6_H_5_, R_5_ = H (**2d**)	**2d′**	1 h	87%
14^*d*^	R_3_ = R_4_ = 4-Me-C_6_H_4_, R_5_ = H (**2e**)	**1e′**	1 h	85%
15	R_1_ = C_10_H_21_, R_2_ = H (**1j**)	**1j′**	N.D.	N.D.
16^*d*^	R_3_ = C_10_H_21_, R_4_ = R_5_ = H (**2f**)	**1j′**	N.D.	N.D.

^*a*^Conditions: alkyne derivatives (0.5 mmol, 0.125 M), [Ca_2_N]^+^·e^–^ (2.5 mmol, 5 equiv.), r.t.

^*b*^Isolated yield.

^*c*^HMPA : iPrOH (v/v = 1 : 1) was used instead of DMF : iPrOH (v/v = 1 : 1).

^*d*^Conditions: alkene derivatives (0.5 mmol, 0.125 M), [Ca_2_N]^+^·e^–^ (1.5 mmol), DMF : MeOH (v/v = 1 : 1), r.t.

It was found that no reaction occurred with the use of aliphatic substrates such as 1-dodecyne **1j** and 1-dodecene **2f** (Table 3, entries 15–16). To clarify the different reactivity between aromatic and aliphatic substrates in thermodynamic considerations, we measured reduction potentials by using cyclic voltammetry (CV), in which the potentials of diphenylacetylene **1a** and 1-dodecyne **1j** were measured by employing a glassy carbon electrode, an Ag/AgCl reference, and a platinum wire auxiliary electrode in a 0.5 M sulphuric acid solution. It was revealed that the reduction potential (–1.54 V *vs.* Ag/AgCl electrode) of **1a** was higher than that (–1.70 V *vs.* Ag/AgCl electrode) of **1j**, indicating that **1a** is a better electron acceptor than **1j**. This result implies that the reduction potential of electrons participating in the reduction of substrates ranges between –1.54 and –1.70 V. This thermodynamic consideration indicates that an appropriate choice of electron transfer media is a critical factor to provoke the electride-mediated reactions *via* the control of reduction potential, since the electrons released from the [Ca_2_N]^+^·e^–^ electride are transferred to substrates through the transfer media.

**Fig. 1:**
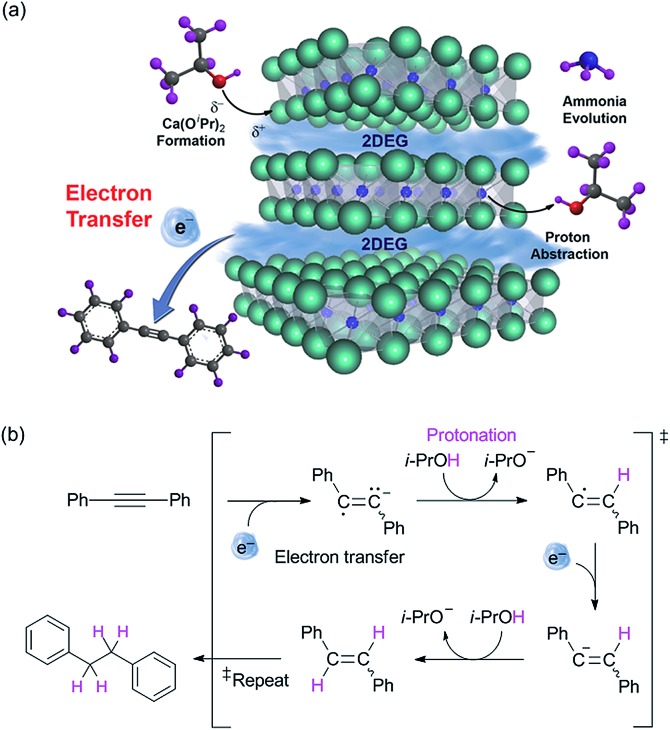
(a) Schematic illustration of the electron transfer mechanism. (b) The proposed mechanism of transfer hydrogenation utilizing electride as the electron transfer agent.

Next, we conducted additional experiments to verify the validity of direct electron transfer *via* the alcoholysis of [Ca_2_N]^+^·e^–^. The decomposition of the electride was confirmed by X-ray diffraction (XRD) measurements and ion-chromatography (IC) that revealed the formation of Ca(O^i^Pr)_2_ and ammonia, respectively (ESI†). The reaction of diphenylacetylene was conducted in the presence of radical scavengers under optimized conditions to clarify the radical reaction that occurs by donation of electrons from the electride *via* the single electron transfer (SET) process. The employment of various radical scavengers, such as TEMPO, 1,4-dinitrobenzene and galvinoxyl, decreased the yields of **1a′** to a level corresponding to the amount of scavengers (see ESI†). On the basis of these results, we proposed a plausible mechanism that involves the SET process (Fig 1). In this process, the use of isopropanol eliminated the need for an additional hydrogen source.

**Fig. 2:**
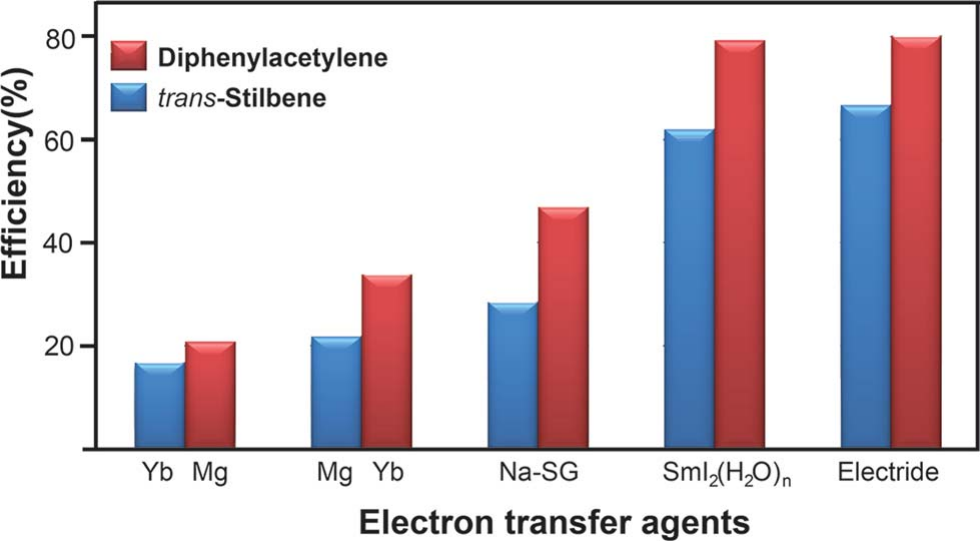
Comparison of electron transfer efficiency in the reduction of alkyne (red) and alkene (blue) using a typical electron transfer agent and [Ca_2_N]^+^·e^–^ electride. The Na-SG is the sodium metal in the silica gel.

Finally, we compared the electron transfer efficiency of the electride-mediated reactions to the efficiency of reactions using various electron transfer agents (Fig. 2). The present reaction using [Ca_2_N]^+^·e^–^ electrides showed a higher electron transfer efficiency than that of other agents [Yb, Mg, Na-SG, SmI_2_(H_2_O)_*n*_], indicating that this protocol is simple and efficient for the reduction of C–C multiple bonds. In comparison with other agents, the electron transfer efficiency for the [Ca_2_N]^+^·e^–^ electride was significantly higher than that for Yb, Mg, and Na-SG, and similar to the costly compound SmI_2_(H_2_O)_*n*_. Furthermore, we estimated the electron transfer efficiency of all electride-mediated organic syntheses (ESI†). The present transfer hydrogenation showed a better performance than the other reactions using [Ca_2_N]^+^·e^–^ and C12A7:e^–^ electrides such as pinacol coupling^12^ and hydrotrifluoromethylations,^14^ supporting the simplicity and effectiveness of the reaction protocol.

## Conclusions

We have developed a simple and highly efficient transfer hydrogenation process utilizing a [Ca_2_N]^+^·e^–^ electride as an electron donor in an alcoholic solvent, as a hydrogen atom donor. This methodology provides a new class of materials as a promising alternative for reducing the processes of C–C multiple bonds in terms of high electron transfer efficiency using an electride composed of naturally abundant elements.

## Supplementary Material

Supplementary information
