# Supplementary material for: Two dimensional inorganic electride-promoted electron transfer efficiency in transfer hydrogenation of alkynes and alkenes
Source: Chem Sci. 2015 Apr 13;6(6):3577–81. doi: 10.1039/c5sc00933b (PMC5659176; doi:10.1039/c5sc00933b)
Supplement: Supplementary file 1 [file SC-006-C5SC00933B-s001.pdf]

Electronic Supplementary Material (ESI) for Chemical Science.
This journal is © The Royal Society of Chemistry 2015

Supporting Information

Two-dimensional electride – promoted electron transfer efficiency in transfer hydrogenation of alkynes and alkenes

Ye Ji Kim,[a,b‡] Sun Min Kim,[a‡] Eun Jin Cho,[c] Hideo Hosono,[d] Jung Woon Yang,[a*] Sung Wng Kim[a,b*]

[a] Department of Energy Science, Sungkyunkwan University, Suwon 440-746, Republic of Korea.
[b] IBS Centre for Integrated Nanostructure Physics, Institute for Basic Science, Suwon 440-746, Republic of Korea
[c] Department of Chemistry, Chung-Ang University, Seoul 156-756, Republic of Korea.
[d] Materials and Structures Laboratory, Tokyo Institute of Technology, Yokohama 226-8503, Japan
‡These authors contributed equally to this work.

Contents

General Methods **S2**
Characterization Data for Products **S3**
^{1}H NMR and ^{13}C NMR Spectra of the Products **S7**
Electron transferring Efficiency Calculation **S18**
Mechanistic Studies **S19**
References **S22**

General Methods

^{1}H NMR spectra were recorded on a Varian at 500 MHz in $CDCl_3$ (δ 7.26 ppm), ^{13}C NMR spectral measurements were performed at 125 MHz using $CDCl_3$ (δ 77.16 ppm). The terms m, s, d, t, q, quint., and sept. represent multiplet, singlet, doublet, triplet, quadruplet, quintuplet, and septet, respectively, and the term br means a broad signal.
The measurement of X-ray diffraction patterns for $Ca(O^iPr)_2$ was made over a 2θ range from 5° to 40° along with a step size of 0.02° and scanning speed was set at 1° at 1°min^{-1} with filtered Cu K_α radiation λ=0.15418 nm (Rigaku Smart Lab, Japan).
Ion chromatography was performed on Metrohm 833 IC plus with conductivity detector (solvent: 1.7mM HNO_3 + 0.7mM PDCA in DI water, temperature: room temperature, fluent speed : 0.9mL/min). Analytic sample was prepared by the following procedure: Each sample was taken from the individual reaction mixture using micro-glass filters. To make more accurate analysis, it diluted with deionized water (200 times less than in the original sample).

Synthesis of dicalcium nitride $[Ca_2N]^+{\cdot}e^-$ electride

A stoichiometric polycrystalline dicalcium nitride ($[Ca_2N]^+{\cdot}e^-$) was synthesized by the solid-state reaction of calcium nitride(Ca_3N_2) powders and calcium metals. Mixture of Ca_3N_2 powders and calcium chips at a molar ratio of 1:1 were pressed into a pellet form under pressure (20~30 MPa). The pellet was fully covered with molybdenum foil and annealed at 800 ^{o}C for 48 hrs under vacuum (~10^{-3} Pa). Then, the sample was quenched into water. To improve homogeneity of dicalcium nitride $[Ca_2N]^+{\cdot}e^-$, the synthesized sample was ground into a powder in an agate mortar in nitrogen-filled glovebox and re-annealed under the same conditions.

General procedure for transfer hydrogenation of aromatic alkynes

Dicalcium nitride $[Ca_2N]^+{\cdot}e^-$ (235 mg, 2.5 mmol) was added to a suspension of alkyne (0.5 mmol) in 4 ml of dry DMF and *i*PrOH in 1:1 mixture at room temperature. The reaction was stirred until color change of electride(black → white) indicated complete consumption of the starting material, and then the reaction mixture was quenched with water and 5% HCl solution in water and, extracted with Et_2O (15 mL×3), and remove the DMF by washing with DI water. The combined organic layers were dried over $MgSO_4$ and concentrated under vacuum.

General procedure for transfer hydrogenation of aromatic alkenes

Dicalcium nitride $[Ca_2N]^+{\cdot}e^-$ (144 mg, 1.5 mmol) was added to a solution of alkene (0.5 mmol) in 4 mL of dry DMF and MeOH in 1:1 mixture at room temperature. The reaction was stirred until color change of electride from black to white, indicating that the starting material was completely consumed. Upon completion of the reaction, the reaction mixture was quenched with water and washed with aqueous 5% HCl solution. The reaction mixture was extracted with Et_2O (15 mL × 3) and saturated brine. The combined extracts were dried over anhydrous $MgSO_4$, filtered, and concentrated *in vacuo*. The residue was purified by flash column chromatography on silica gel to furnish the corresponding product.

Characterization Data for Products

1,2-diphenylethane (Table 3, Entry 1, 10, 11)

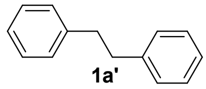

1a'

The physical and spectral data were identical to those previously reported for this compound.[1]

^{1}H NMR (500 MHz, $CDCl_3$); 7.29-7.25(m, 4H), 7.19-7.17(m, 6H), 2.92(s, 4H) ; ^{13}C NMR (125 MHz, $CDCl_3$) δ: 141.9, 128.5, 128.4, 126.0, 38.0 ppm.

1-methyl-2-(phenylethynyl)benzene (Table 3, Entry 2)

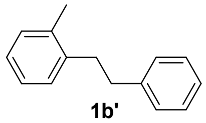

1b'

The physical and spectral data were identical to those previously reported for this compound.[2]

^{1}H NMR (500 MHz, $CDCl_3$); 7.30-7.27(m, 2H), 7.21-7.19(m, 3H), 7.14-7.11(m, 4H), 2.89-2.86(m, 4H), 2.94(s, 3H) ; ^{13}C NMR (125 MHz, $CDCl_3$) δ: 142.1, 140.1, 136.0, 130.3, 128.9, 128.5, 126.2, 126.1, 126.0, 36.8, 35.5, 19.3 ppm.

1-methoxy-3-phenethylbenzene (Table 3, Entry 3)

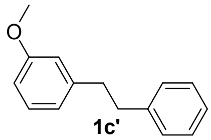

1c'

The physical and spectral data were identical to those previously reported for this compound.[3]

^{1}H NMR (500 MHz, $CDCl_3$); 7.29-7.26(m, 2H), 7.21-7.17(m, 4H), 6.79(d, J = 10 Hz, 1H), 6.75-6.71(m, 2H), 3.76(s, 3H), 2.90(br s, 4H) ; ^{13}C NMR (125 MHz, $CDCl_3$) δ: 159.6, 143.5, 141.8, 129.3, 128.5, 128.4, 126.0, 120.9, 114.2, 111.3, 55.1, 38.0, 37.9 ppm.

1-methoxy-4-phenethylbenzene (Table 3, Entry 4)

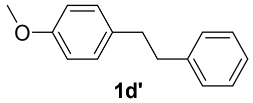

1d'

The physical and spectral data were identical to those previously reported for this compound.[2]

^{1}H NMR (500 MHz, $CDCl_3$); 7.27-7.24(m, 2H), 7.17-7.15(m, 3H), 7.08-7.06(m, 2H), 6.81-6.80(m, 2H), 3.75(s, 3H) 2.87-2.83(m, 4H) ; ^{13}C NMR (125 MHz, $CDCl_3$); 157.9, 141.9, 134.0, 129.4, 128.6, 128.6, 126.0, 126.0, 113.8, 113.8, 55.3, 38.3, 37.1 δ: ppm.

1,2-di-*p*-tolylethane (Table 3, Entry 5, 13)

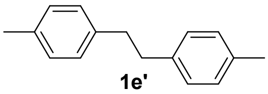

1e'

The physical and spectral data were identical to those previously reported for this compound.[4]

^{1}H NMR (500 MHz, $CDCl_3$); 7.14(s, 8H), 2.91(s, 4H), 2.37(s, 6H) ; ^{13}C NMR (125 MHz, $CDCl_3$); 138.9, 135.3, 129.1, 128.4, 37.7, 21.1 δ: ppm.

1-fluoro-4-phenethylbenzene (Table 3, Entry 6)

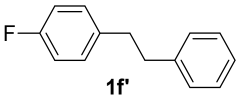

1f'

The physical and spectral data were identical to those previously reported for this compound.[5]

^{1}H NMR (500 MHz, $CDCl_3$); 7.29-7.23(m, 2H), 7.20-7.17(m, 1H), 7.15-7.08(m, 4H), 6.96-6.93(m, 2H), 2.88(s, 4H) ; ^{13}C NMR (125 MHz, $CDCl_3$) δ: 162.3(d, J_F = 242.5 Hz), 141.5, 137.3(d, J_F = 2.5 Hz), 129.9(d, J_F = 7.5 Hz), 128.5, 128.4, 126.0, 115.1(d, J_F = 21.25 Hz), 38.1, 37.1 ppm.

2,4-difluoro-1-phenethylbenzene (Table 3, Entry 7)

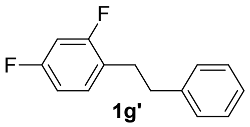

1g'

^{1}H NMR (500 MHz, $CDCl_3$); 7.28-7.26(m, 2H), 7.20-7.14(m, 3H), 7.05-7.00(m, 1H), 6.78-6.72(m, 2H), 2.91-2.85(m, 4H) ; ^{13}C NMR (125 MHz, $CDCl_3$) δ: 162.46 (d, J_F = 12.5Hz), 161.98 (d, J_F = 12.5 Hz), 141.22, 131.18 (dd, J_F = 8.8, 7.5 Hz), 128.50, 128.41, 126.12, 124.29 (dd, J_F = 16.3, 3.8 Hz), 103.64 (t, J_F = 25 Hz), 36.49, 30.68 (d, J_F = 1.3 Hz) ppm. HR-MS(EI) *m/z* calcd for $C_{14} H_{12}F_2$ 218.0907, founded 218.0902.

1,2-bis(4-fluorophenyl)ethane (Table 3, Entry 8)

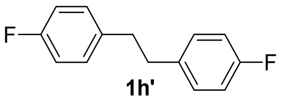

1h'

The physical and spectral data were identical to those previously reported for this compound.[1]

^{1}H NMR (500 MHz, $CDCl_3$); 7.08-7.05(m, 4H), 6.96-6.92(m, 4H), 2.85(s, 4H) ; ^{13}C NMR (125 MHz, $CDCl_3$) δ: 162.3(d, J_F = 242.5 Hz), 137.0(d, J_F = 2.5 Hz), 129.9(d, J_F = 20.0 Hz), 115(d, J_F = 21.25 Hz), 37.2 ppm.

4-ethyl-1,1'-biphenyl (Table 3, Entry 9)

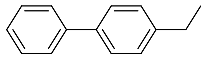

1i'

The physical and spectral data were identical to those previously reported for this compound.[6]

^{1}H NMR (500 MHz, $CDCl_3$); 7.58-7.57(m, 2H), 7.51(d, J = 8.2 Hz, 2H), 7.41(t, J = 7.7 Hz, 2H), 7.33-7.30(m, 1H), 7.28(d, J = 10 Hz, 2H), 2.69(q, J = 7.6 Hz, 2H), 1.27(t, J = 7.6Hz, 3H) ; ^{13}C NMR (125 MHz, $CDCl_3$) δ: 143.4, 141.23, 138.7, 128.8, 128.3, 127.1, 127.1, 127.0, 28.6, 15.7 ppm.

Ethane-1,1,2-triyltribenzene (Table 3, Entry 12)

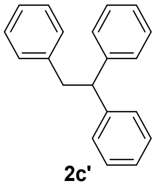

2c'

The physical and spectral data were identical to those previously reported for this compound.[7]

^{1}H NMR (500 MHz, $CDCl_3$); 7.23-7.18(m, 8H), 7.15-7.10(m, 5H), 6.99-6.97(m, 2H), 4.21(t, J = 7.8 Hz, 1H), 3.34(d, J = 7.8 Hz, 2H) ; ^{13}C NMR (125 MHz, $CDCl_3$); 144.6, 140.4, 129.2, 128.5, 128.2, 126.3, 126.0, 53.2, 42.2 δ: ppm.

1,3-dimethoxy-5-phenethylbenzene (Table 3, Entry 13)

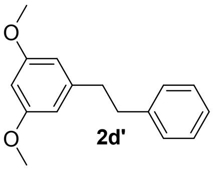

2d'

The physical and spectral data were identical to those previously reported for this compound.[8]

^{1}H NMR (500 MHz, $CDCl_3$); 7.29-7.26(m, 2H), 7.20-7.18(m, 3H), 6.34-6.33(m, 2H), 6.31-6.30(m, 1H), 3.74(s, 6H), 2.92-2.83(m, 4H) ; ^{13}C NMR (125 MHz, $CDCl_3$); 160.8, 144.2, 141.8, 128.5, 128.4, 126.0, 106.6, 98.0, 55.3, 38.3, 37.8 δ: ppm.

1,2-diphenylethane (Table 3, Entry1, 10, 11)

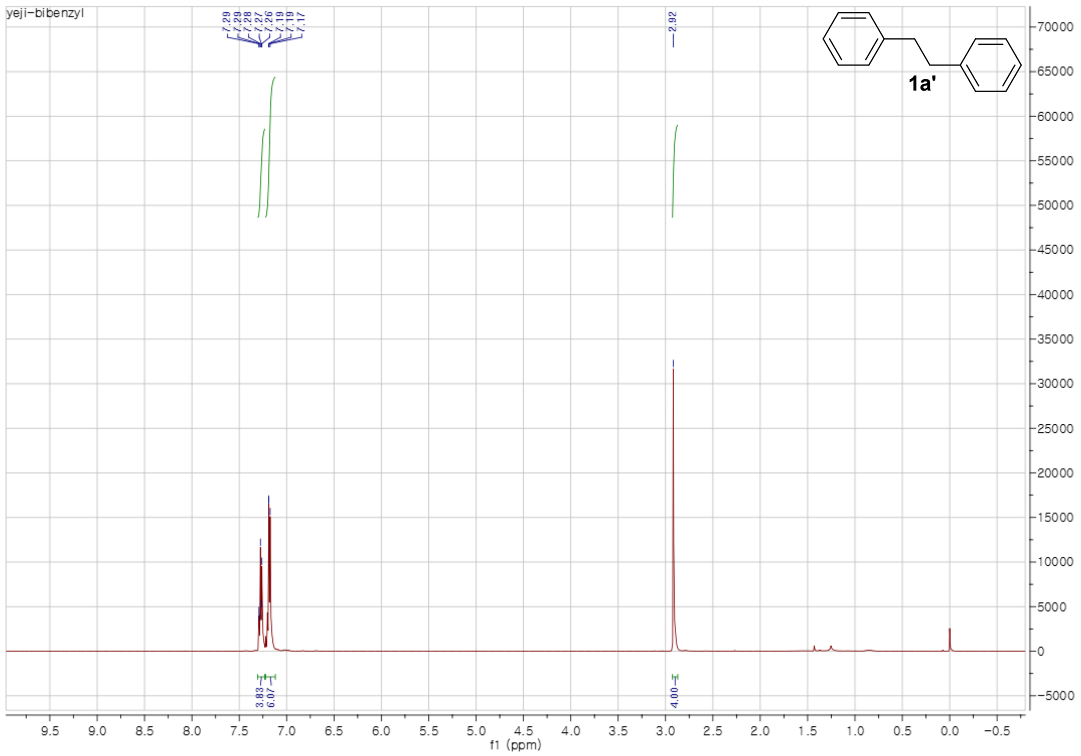

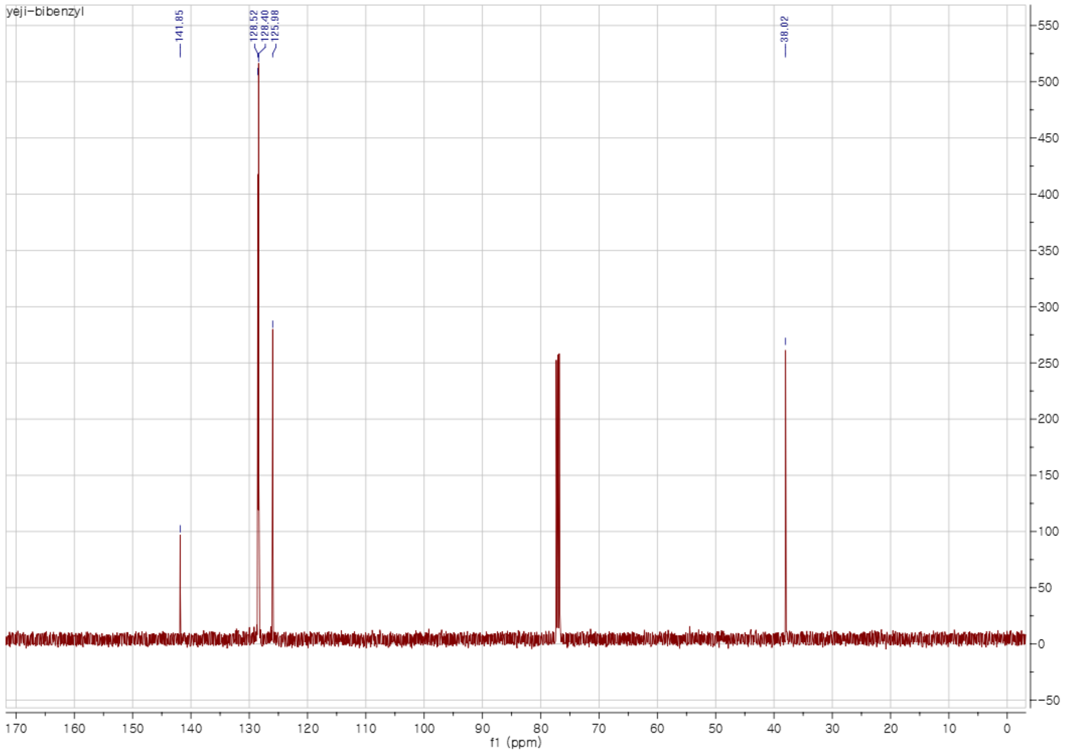

1-methyl-2-(phenylethynyl)benzene (Table 3, Entry 2)

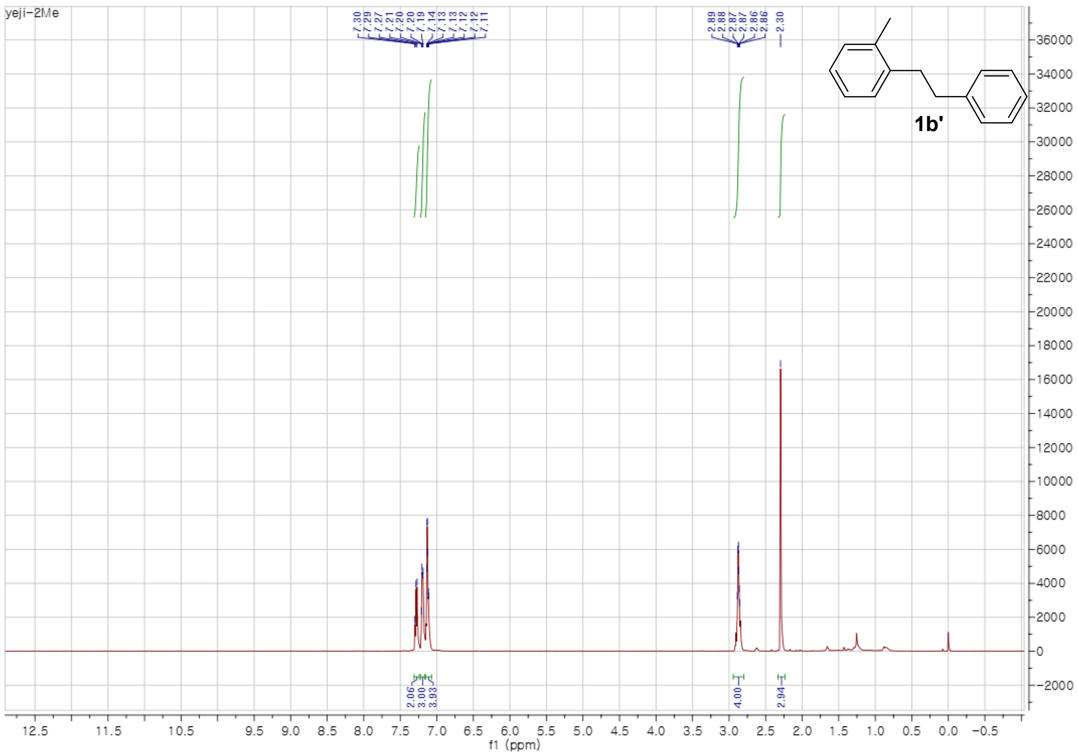

.

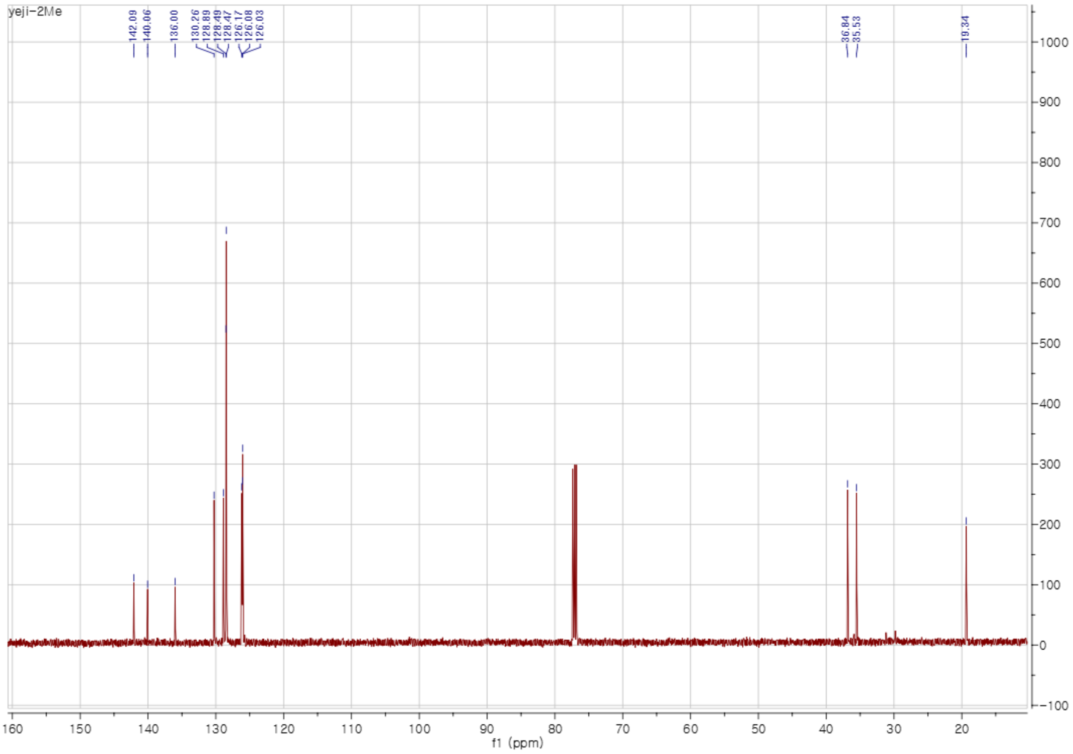

1-methoxy-3-phenethylbenzene (Table 3, Entry 3)

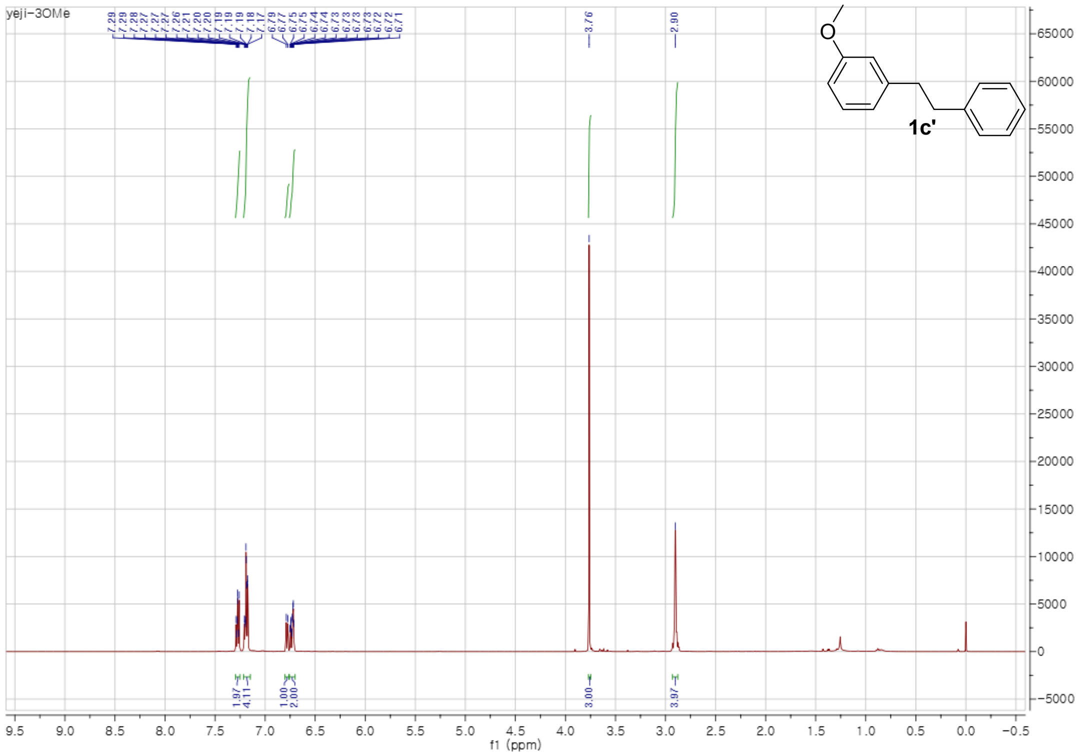

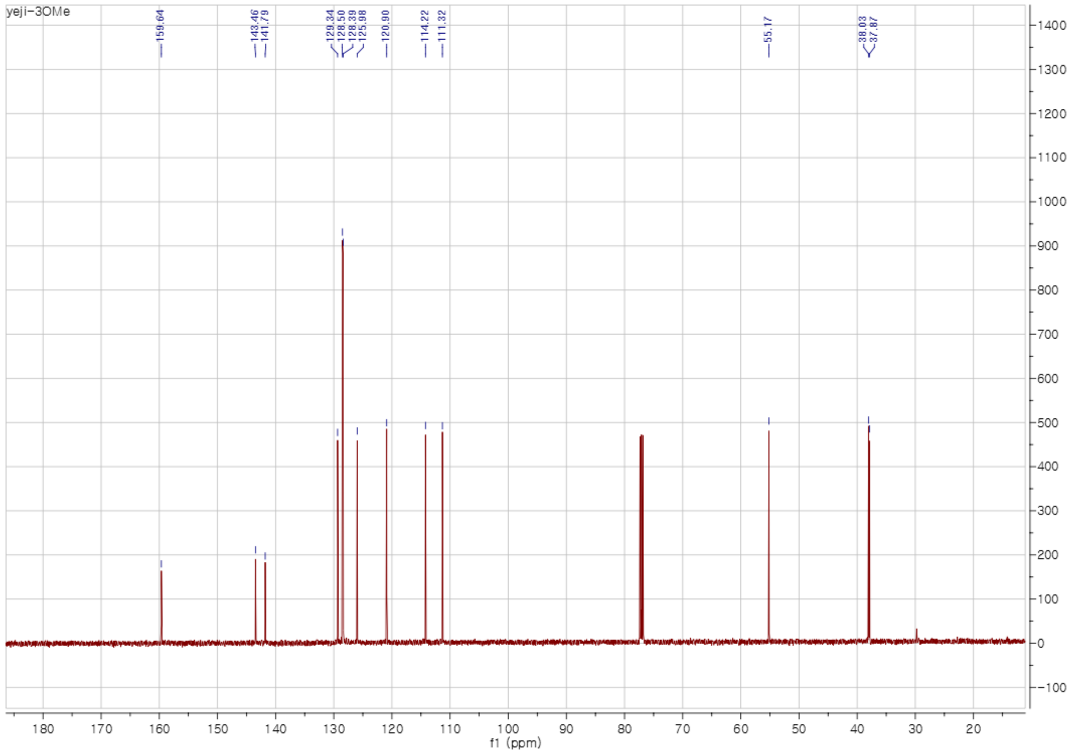

1-methoxy-4-phenethylbenzene (Table 3, Entry 4)

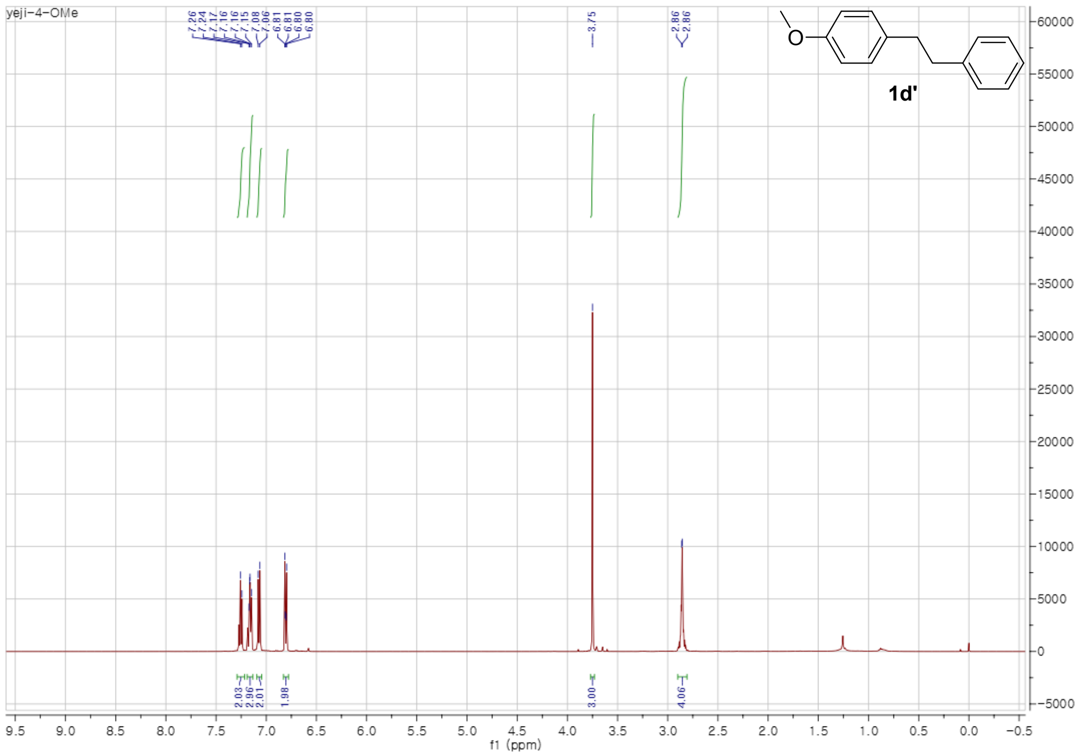

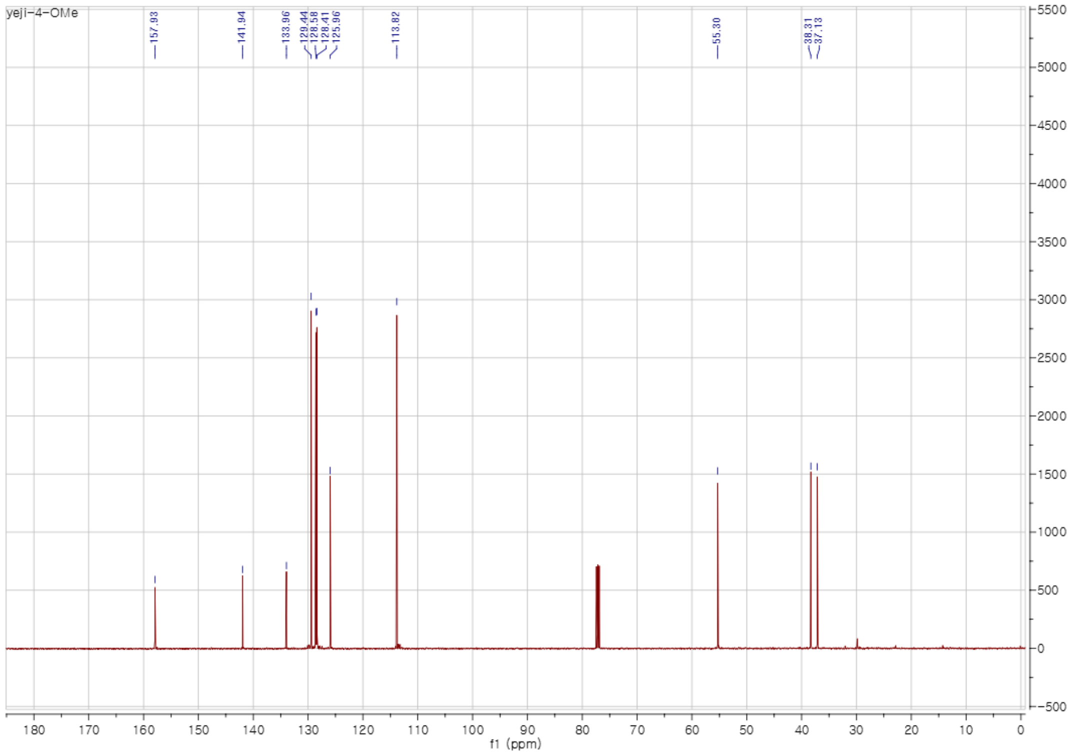

1,2-di-p-tolylethane (Table 3, Entry 5, 13)

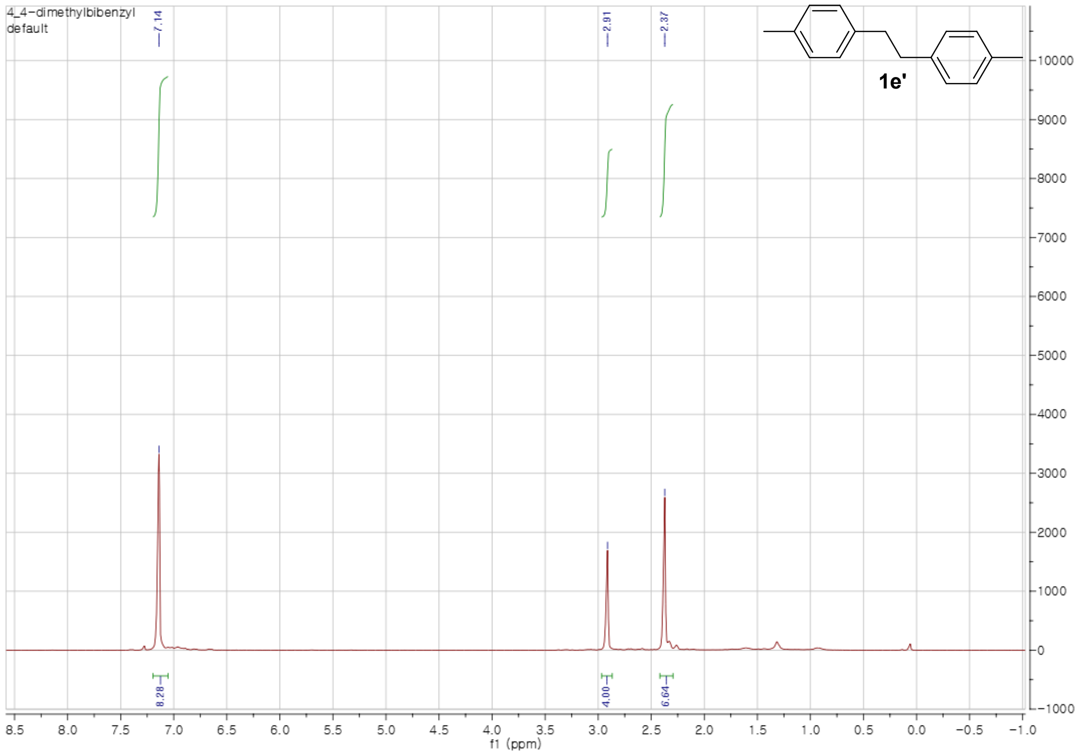

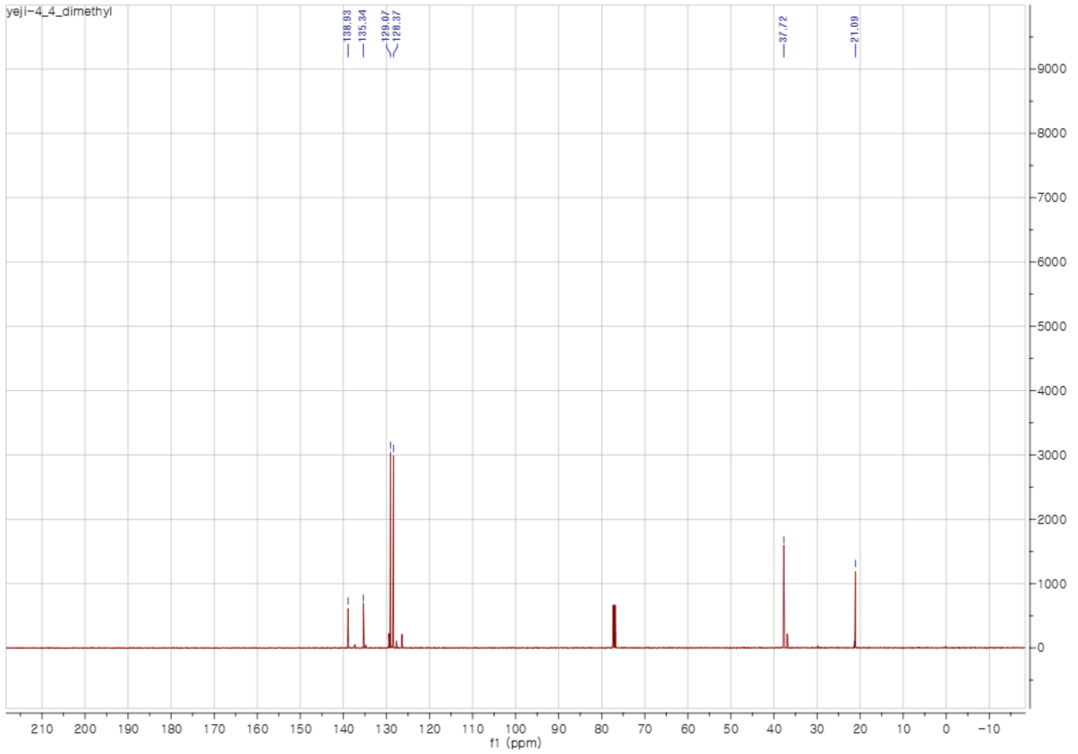

1-fluoro-4-phenethylbenzene (Table 3, Entry 6)

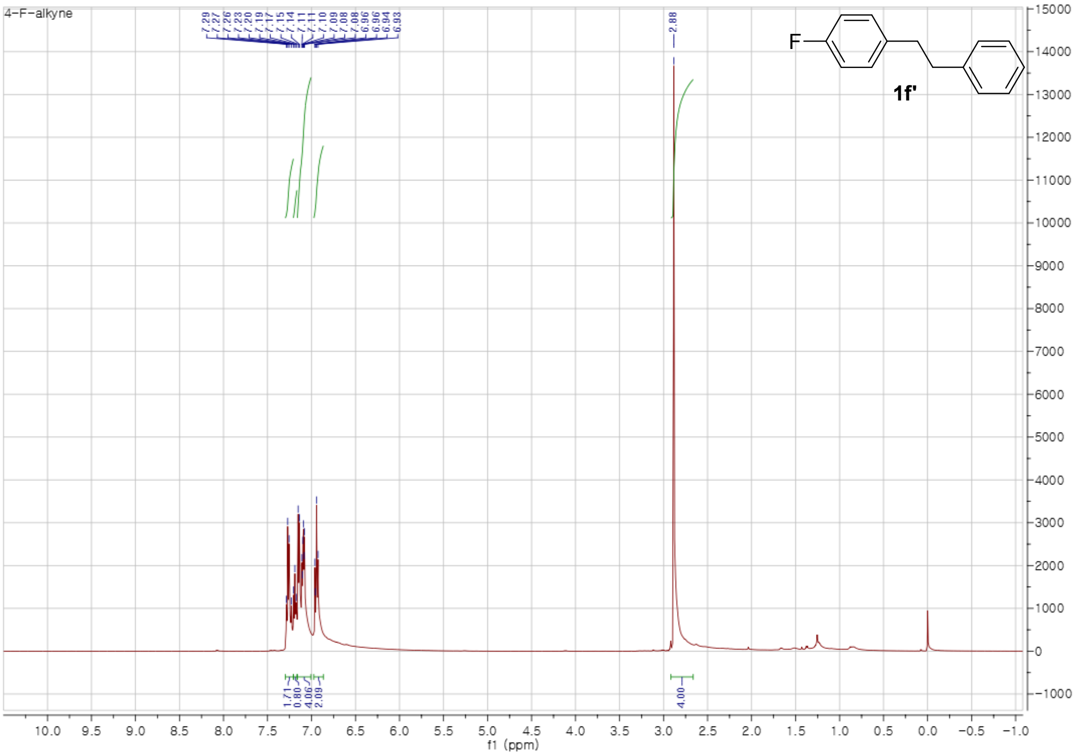

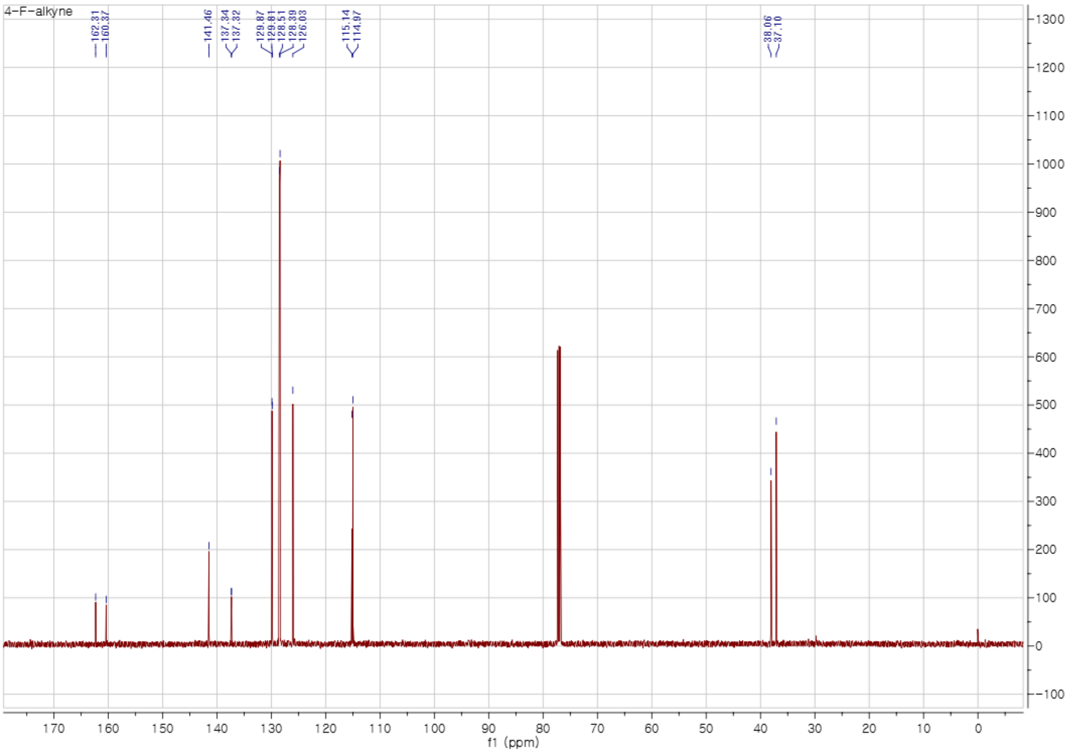

2,4-difluoro-1-phenethylbenzene (Table 3, Entry 7)

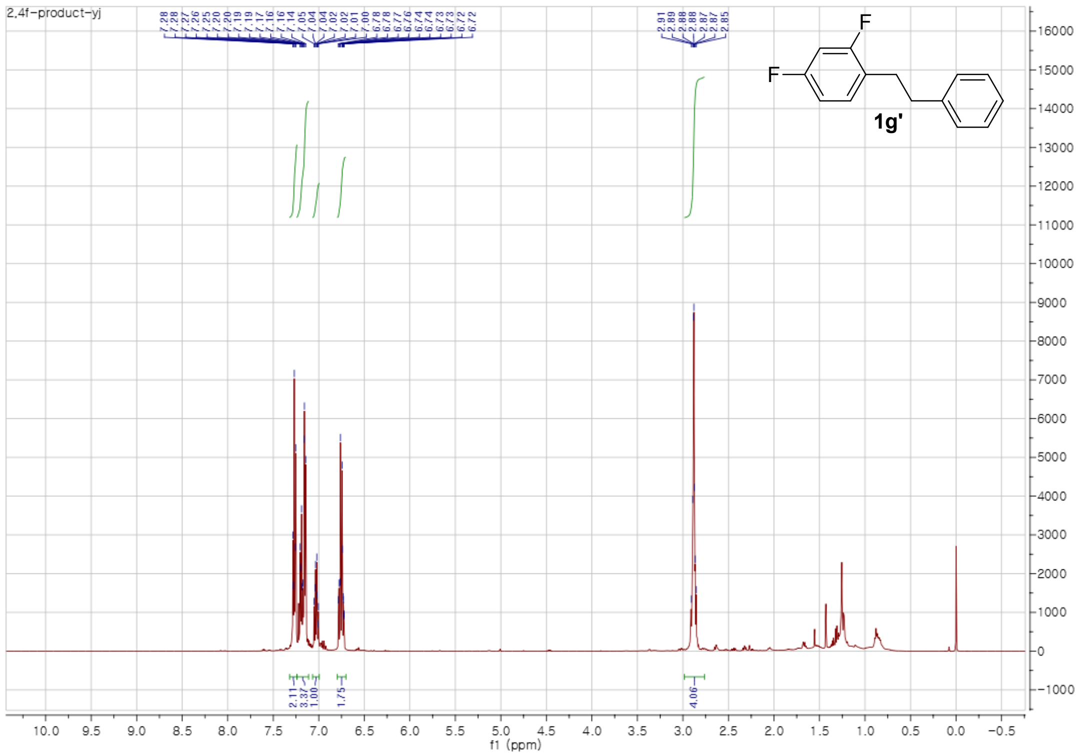

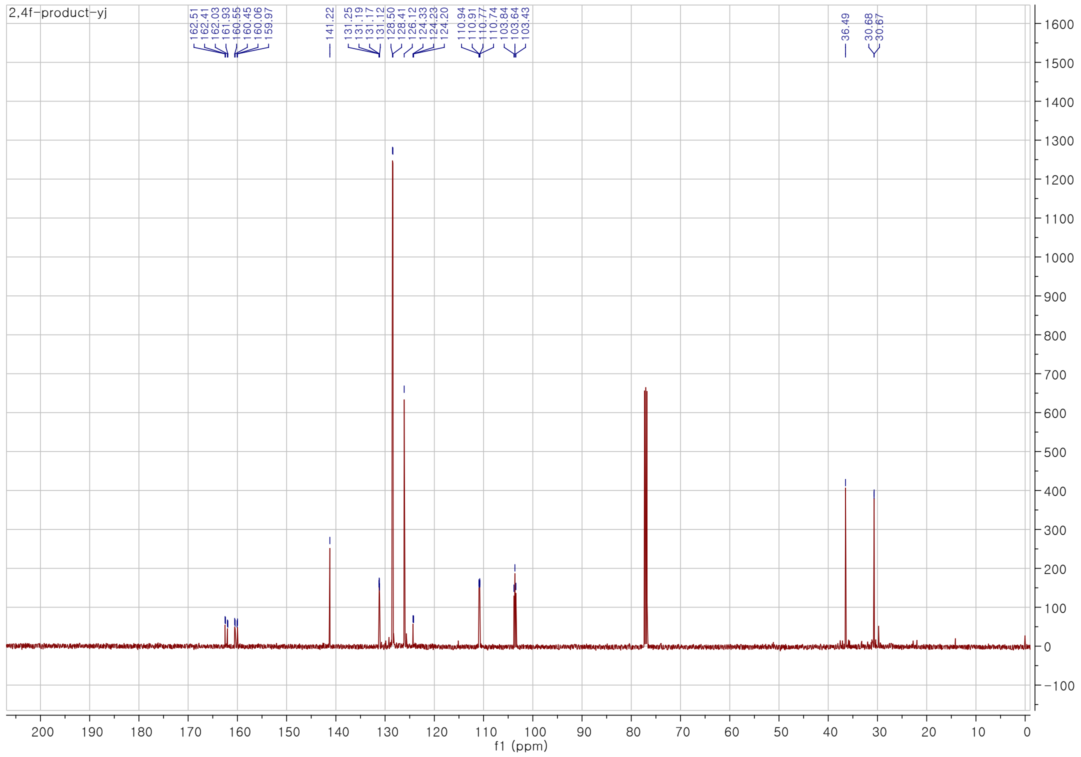

.1,2-bis(4-fluorophenyl)ethane (Table 3, Entry 8)

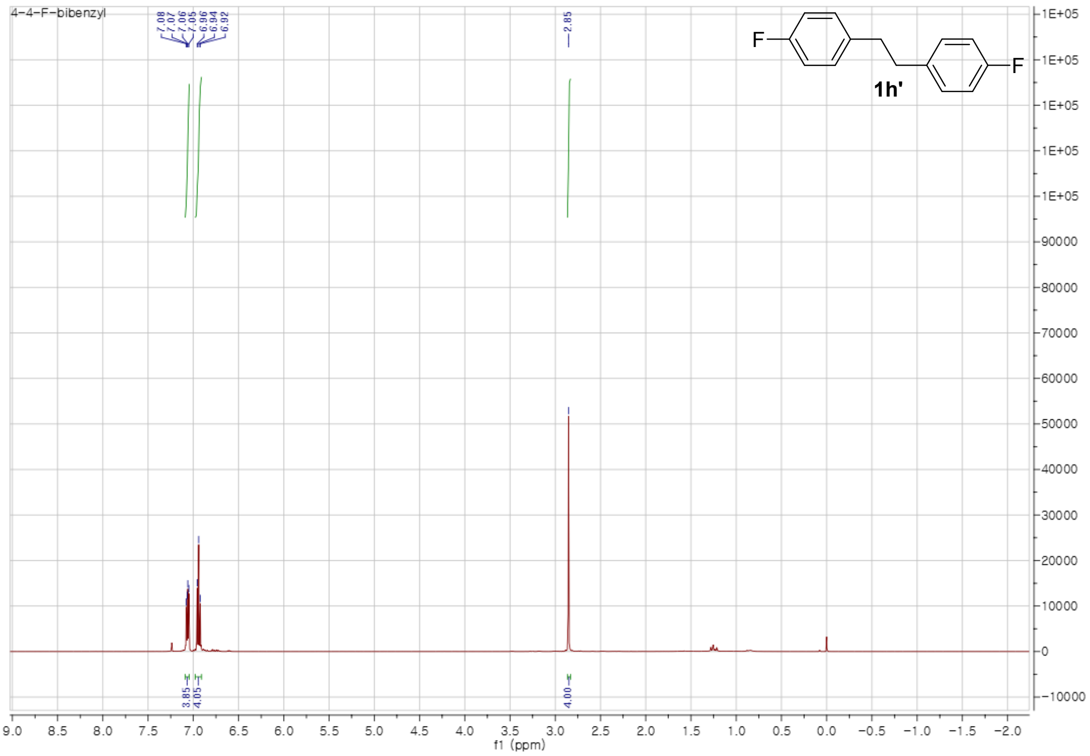

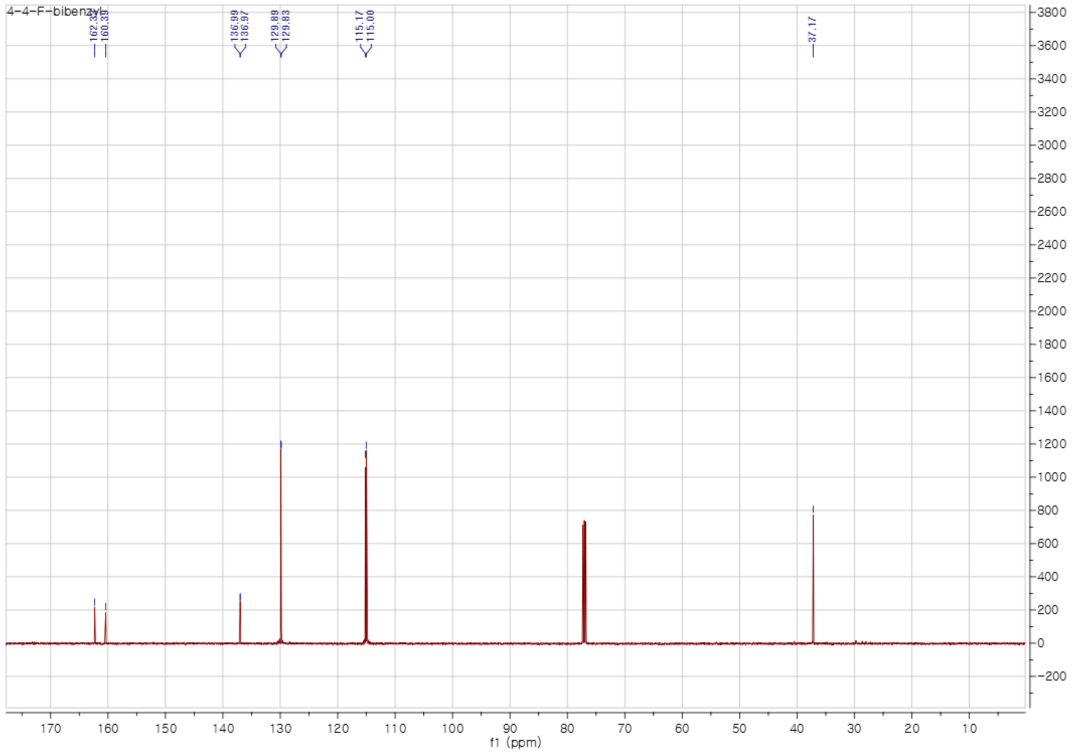

4-ethyl-1,1'-biphenyl (Table 3, Entry 9)

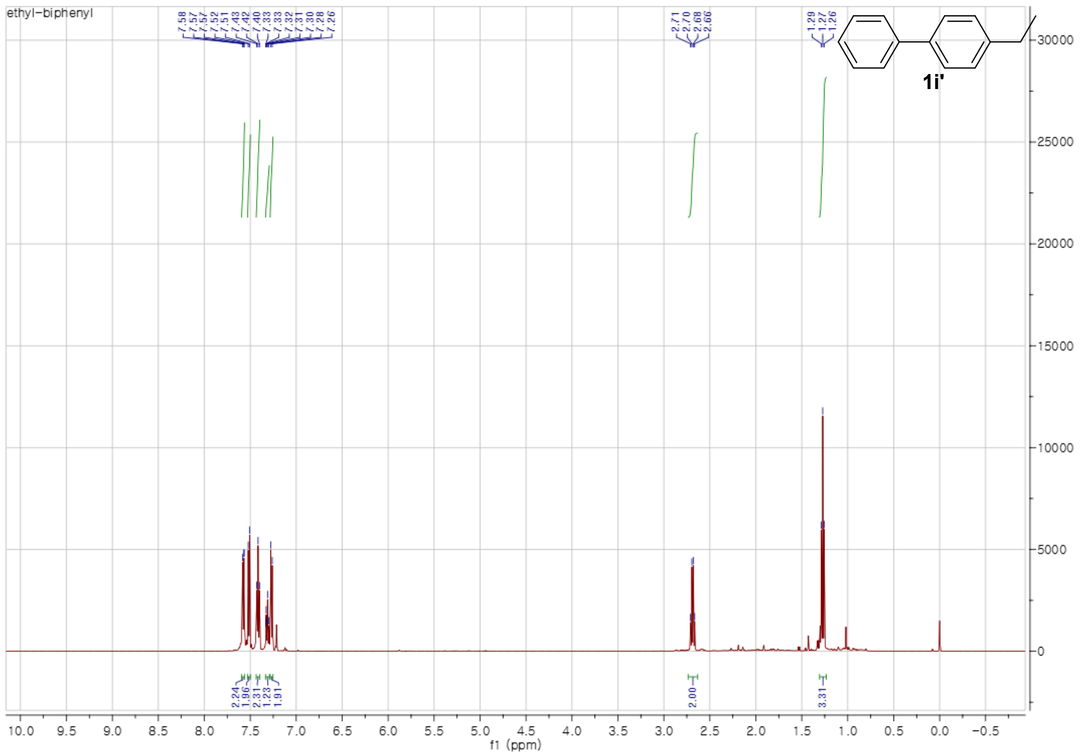

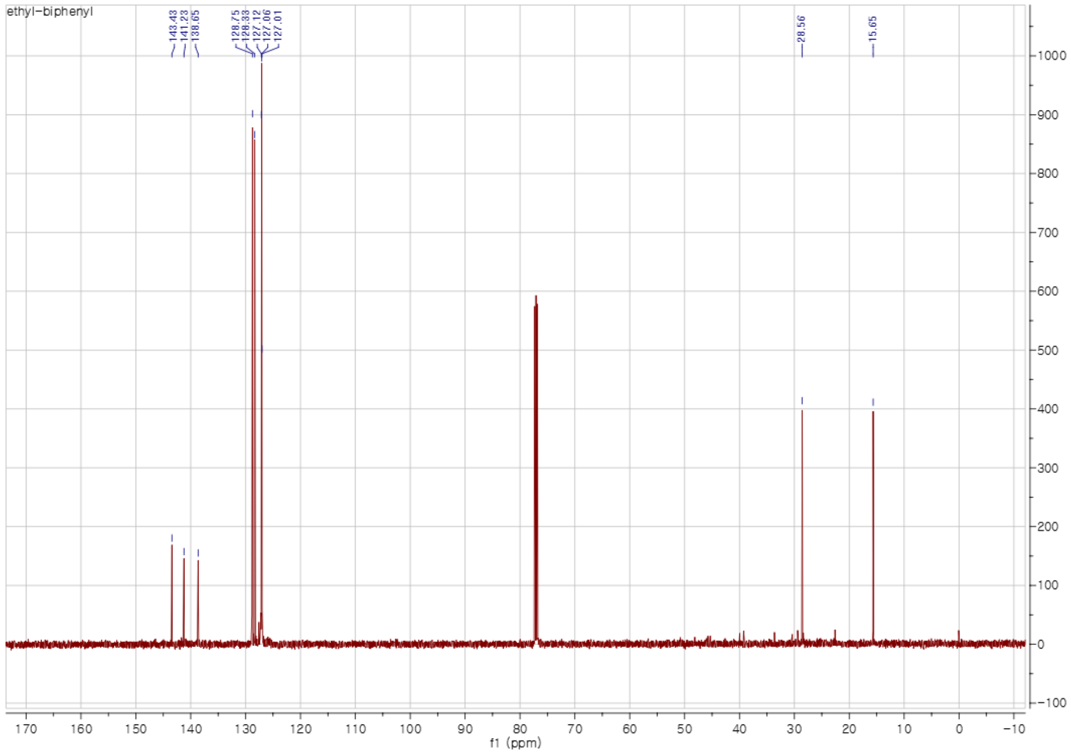

Ethane-1,1,2-triyltribenzene (Table 3, Entry 12)

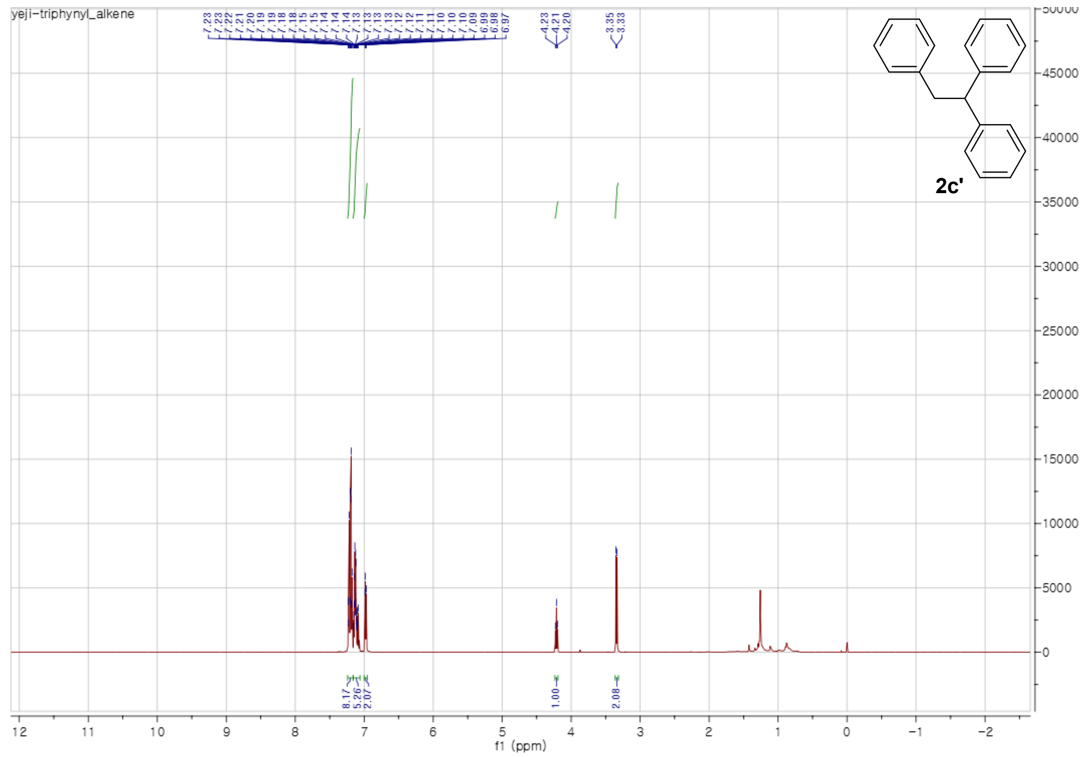

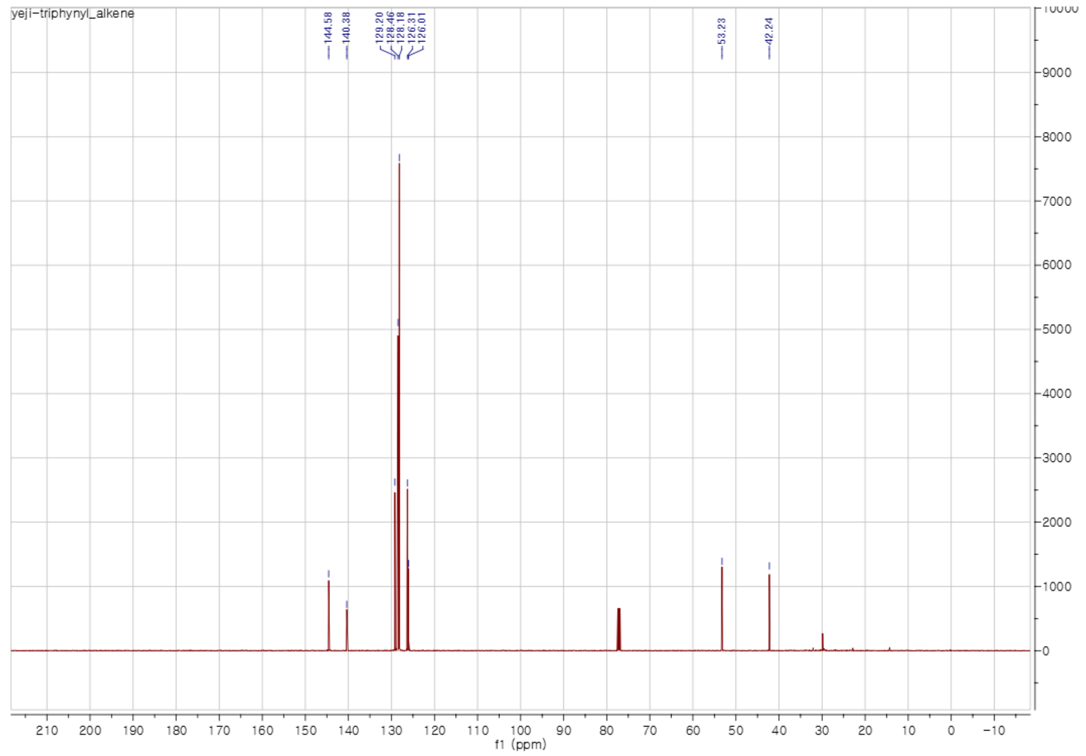

1,3-dimethoxy-5-phenethylbenzene (Table 3, Entry 13)

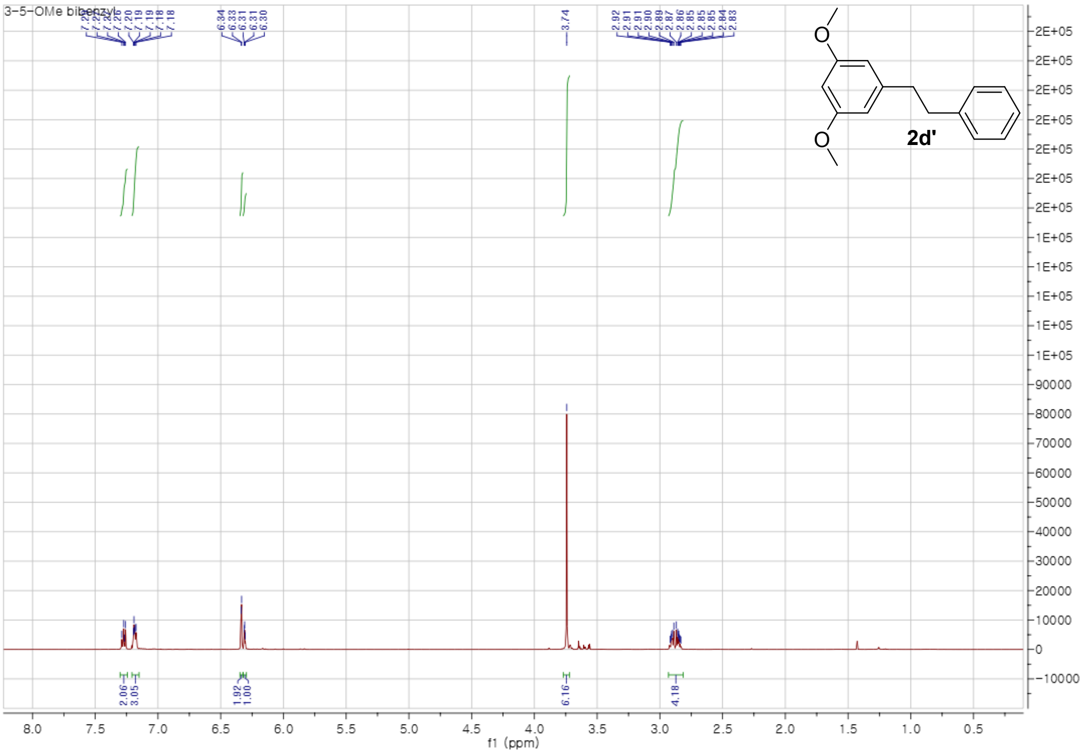

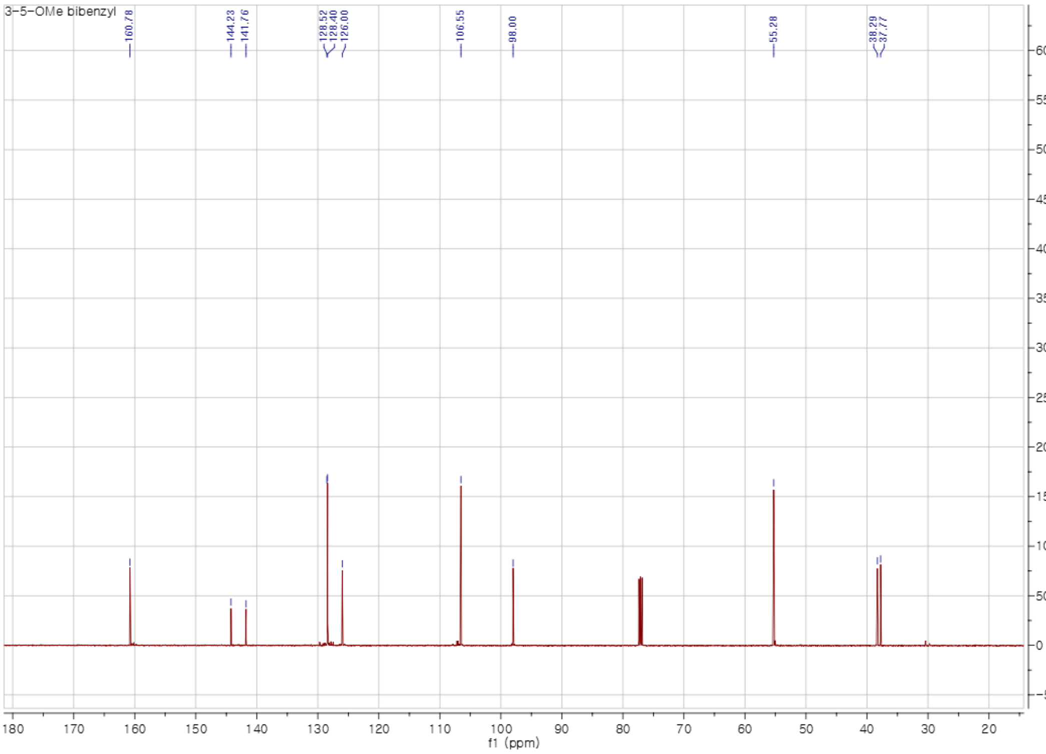

Electron Transferring Efficiency Calculation [9-16]

Efficiency = [participated electron (equiv.) / provided electron (equiv.)] x 100 (%)

Reagent	Participated electron (equiv.)	Provided electron (equiv.)	Efficiency (%)
Yb^{3+}	1.9	9	21
Mg^{2+}	3.4	10	34
Na-SG	3.3	7	47
SmI_2/H_2O	4	5	79
$[Ca_2N]^+ \cdot e^-$	4	5	80
Reagent	Participated electron (equiv.)	Provided electron (equiv.)	Efficiency (%)
Mg^{2+}	1.7	10	17
Yb^{3+}	2	9	22
Na-SG	2	7	29
SmI_2/H_2O	2	3	62
TmI_2	2	3	66.7
$[Ca_2N]^+ \cdot e^-$	2	3	66.7
Reaction	Participated electron (equiv.)	Provided electron (equiv.)	Efficiency (%)
Pinacol coupling using C12A7	0.7	11.6	6
Pinacol coupling using $[Ca_2N]^+ \cdot e^-$	1	2	50
Hydrotrifluoro methylation using $[Ca_2N]^+ \cdot e^-$	0.9	3	30
Transfer hydrogenation using $[Ca_2N]^+ \cdot e^-$	4	5	80

Mechanistic studies (Employment of radical scavengers)

Table S1. Electrides mediated reduction of diphenylacetylene in the presence of radical scavengers

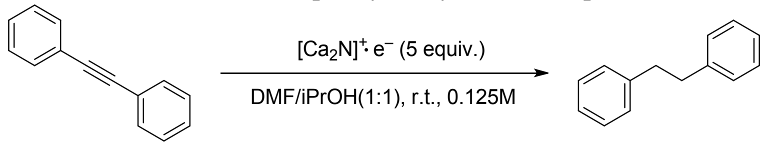

Additive	Time	Equiv.	Yield[a]
**None**	15h	-	99%
**TEMPO**	7h	1eq	64%
**Galvioxynol**	7h	1eq	58%
**1,4-dinitrobenzene**	18h	1eq	48%

[a] determined by GC (Using biphenyl as an internal standard)

Mechanistic studies [X-ray diffraction patterns of $Ca(O^iPr)_2$ and reaction time log]

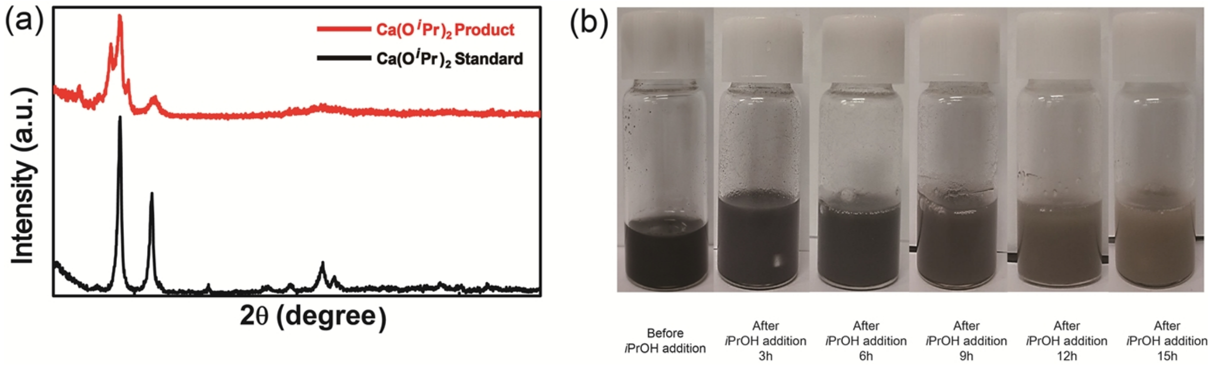

Fig. S1 (a) XRD patterns of $Ca(O^iPr)_2$ standard powders (black line) and resulting $Ca(O^iPr)_2$ product from the reaction mixtures (red line). All measurements are performed under nitrogen atmosphere. (b) Reaction time log; each picture was taken every 3h.

Mechanistic studies [Ion chromatography of NH_4^+ (ammonium ion)]

1) NH_4^+ cation calibration curve

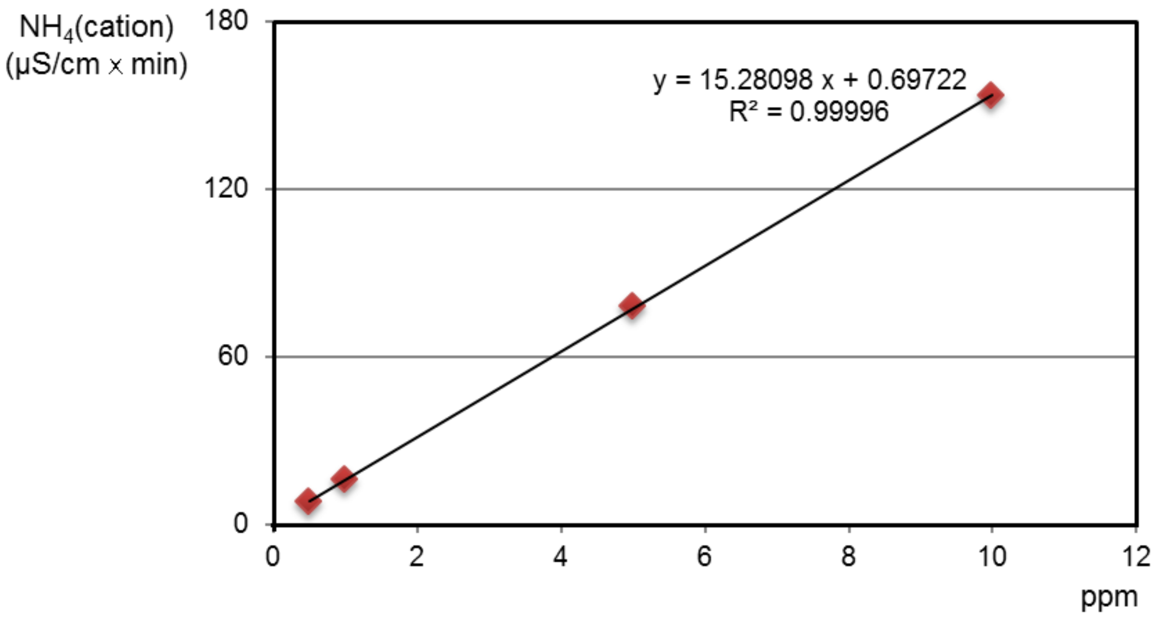

Function: A = 15.28098B+0.69722 × Q
Relative standard deviation: 1.115%
Correlation coefficient: 0.99996

Sample	Conc.(mg/L)	Volume(μL)	Dilution	Area
Standard 1	0.500	1	1.0	8.155
Standard 2	1.000	1	1.0	15.8
Standard 3	5.000	1	1.0	77.77
Standard 4	10.000	1	1.0	153.2

2) Result

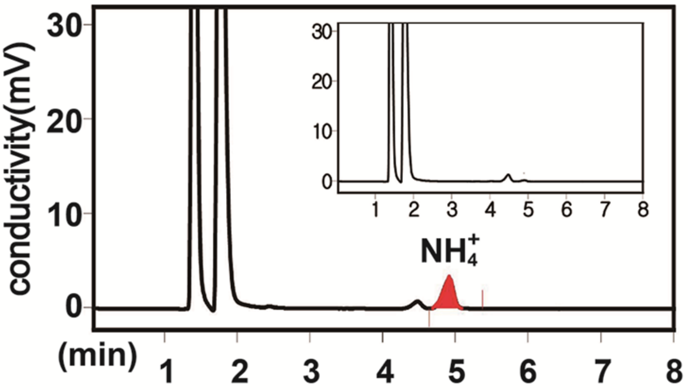

**Sample (THF) - Inset**	
Electride	$[Ca_2N]^+ \cdot e^-$ (235 mg, 2.5 mmol)
Alkyne	diphenylacethylene (90 mg, 0.5 mmol)
Solvent	THF 4 mL
Washed Solvent	THF 8 mL
Dilution	200 times
Pressure / Flow	7.88 MPa / 0.700 mL/min
IC value	2.215 mV
Final concentration	28.4mg/kg
**Sample (THF+*i*PrOH)**	
Electride	$[Ca_2N]^+ \cdot e^-$ (235 mg, 2.5 mmol)
Aldehyde	diphenylacethylene (90 mg, 0.5 mmol)
Solvent	*i*PrOH 2 mL / THF 2 mL
Washed Solvent	*i*PrOH 8 mL
Dilution	200 times
Pressure / Flow	7.88 MPa / 0.700 mL/min
IC value	0.142mV
Final concentration	425.0mg/kg

References

1 G. A. Molander, C. –S. Yun, M. Ribagorda and B. Biolattom, *J. Org. Chem.*, 2003, **68**, 5534.

2 C, T, To, K. S. Choi and K. S. Chan, *J. Am. Chem. Soc.* , 2012, **134**, 11388.

3 L. Zhang, Z. Zuo, X. Leng and Z. Huang, *Angew. Chem.*, 2014, **126**, 2734; *Angew. Chem. Int. Ed.*, 2014, **53**, 2696.

4 K. Sato, Y. Inoue, T. Mori, A. Sakaue, A. Tarui, M. Omote, I. Kunmadali and A. Ando, *Org. Lett.*, 2014, **16**, 3756.

5 J. D. St. Denis, C. C. G. Scully, C. F. Lee, A. and K. Yudin, *Org. Lett.*, 2014, **16**, 1388.

6 A. Modvig, T. L. Andersen, R. H. Tanning, A. T. Lindhardt and T. Skrystrup, *J. Org. Chem.*, 2014, **79**, 5861.

7 M. Szostak, M. Spain and D. J. Procter, *J. Org. Chem.*, 2014, **79**, 2522.

8 M. Blangetti, P. Fleming and D. F. O'Shea, *J. Org. Chem.*, 2012, **77**, 2870.

9 Z. How, H. Taniguchi and Y. Fujiwara, *Chem. Lett.*, 1987, **16**, 305.

10 R. O. Hutchins, R. X. Taffer, I. M. Sivakumar, R. A. Monaghan and E. M. Elisseou, *Tetrahedron Lett.*, 1989, **30**, 55.

11 M. J. Costanzo, M. M. Patel, K. A. Petersen and P. F. Vogt, *Tetrahedron Lett.*, 2009, ***50***, 5463.

12 M. Szostak, M. Spaion and D. J. Procter, *J. Org. Chem.*, 2014, **79**, 2522

13 M. Szostak, M. Spain and D. J. Procter, *Angew. Chem*., 2013, **125**, 7378; *Angew. Chem. Int. Ed.*, 2013, **52**, 7237.

14 H. Buchammagari, Y. Toda, M. Hirano, H. Hosono, D. Takeuchi and K. Osakada, *Org. Lett.*, 2007, **9**, 4287.

15 Y. J. Kim, S. M. Kim, H. Hosono, J. W. Yang and S. W. Kim, *Chem. Commun.*, 2014, **50**, 4791

16 S. Choi, Y. J. Kim, S. M. Kim. J. W. Yang, S. W. Kim and E. J. Cho, *Nat. Commun.*, 2014, **5**, 4481.
